# Live yeast in juvenile diet induces species-specific effects on *Drosophila* adult behaviour and fitness

**DOI:** 10.1038/s41598-019-45140-z

**Published:** 2019-06-20

**Authors:** Juliette Murgier, Claude Everaerts, Jean-Pierre Farine, Jean-François Ferveur

**Affiliations:** Université de Bourgogne Franche-Comté, Centre des Sciences du Goût et de l’Alimentation, AgroSup-UMR 6265 CNRS, UMR 1324 INRA, 6, Bd Gabriel, F-21000 Dijon, France

**Keywords:** Chemical ecology, Behavioural ecology

## Abstract

The presence and the amount of specific yeasts in the diet of saprophagous insects such as *Drosophila* can affect their development and fitness. However, the impact of different yeast species in the juvenile diet has rarely been investigated. Here, we measured the behavioural and fitness effects of three live yeasts (*Saccharomyces cerevisiae* = SC; *Hanseniaspora uvarum* = HU; *Metschnikowia pulcherrima* = MP) added to the diet of *Drosophila melanogaster* larvae. Beside these live yeast species naturally found in natural *Drosophila* populations or in their food sources, we tested the inactivated “drySC” yeast widely used in *Drosophila* research laboratories. All flies were transferred to drySC medium immediately after adult emergence, and several life traits and behaviours were measured. These four yeast diets had different effects on pre-imaginal development: HU-rich diet tended to shorten the “egg-to-pupa” period of development while MP-rich diet induced higher larval lethality compared to other diets. Pre- and postzygotic reproduction-related characters (copulatory ability, fecundity, cuticular pheromones) varied according to juvenile diet and sex. Juvenile diet also changed adult food choice preference and longevity. These results indicate that specific yeast species present in natural food sources and ingested by larvae can affect their adult characters crucial for fitness.

## Introduction

Host-microbe and microbe-microbe interactions can highly impact animal ecology, evolution and behaviour^1–7^. In insects, the effect of fungi has been much less documented than that of bacteria^8,9^, despite the importance of their relationship with regard to fitness^10,11^ and behaviour^12^. While the microbes ingested and hosted in the insect gut can strongly impact the survival of their host^13,14^, their effect on reproduction and speciation is less clear^15–18^. With a relatively limited number of bacterial and fungal species hosted in its gastro-intestinal tract, *Drosophila* is a favourable host-model organism to evaluate their impact^19–21^. Most *Drosophila* species feed on rotting plants and fruits, thus promoting the ingestion of microorganisms which can be released through their feces on spatially distant food sources^22,23^. Specific yeast species (or a mixture of yeast and bacterial species) present on natural food substrates can attract flies of different species of *Drosophila* over greater distance^12,24,25^. In the laboratory, individuals of D. melanogaster have been shown to exhibit olfactory preference for different yeast-rich diets^26^.

At a distance, insects are not only attracted by microbes on food sources, but by the metabolites resulting from the activity of gut-associated microbes^27,28^. These metabolites are mostly released through feces outside the larval digestive tract and some of them can change larval olfactory preference and induce life-long changes in egg-laying and olfactory behaviours in insects “conditioned” with such metabolites^12,29–31^. Such conditioning to specific food components and/or microbial metabolites can induce, through epigenetic modification, a persistent change in food preference across generations^32,33^. This shift, in turn, may allow insects to quickly adapt to new food sources and establish a tight relationship with specific host plants^34^, thus explaining why some pest insects are able to rapidly develop resistance mechanisms against antifungal and/or insecticidal compounds^35^. In several studies on Drosophila, life-long effects due to quantitative variation of yeast in the juvenile diet have been investigated. In particular, a restricted yeast supply during larval development induced increased adult longevity^9,26^, together with body fat content and starvation resistance, while it decreased adult body size and immune gene expression^36–38^. Moreover, the addition of the yeast species *Saccharomyces cerevisiae* subjected to various physical treatments (heat, desiccation) in the juvenile diet altered adult food preference, copulation, fecundity and longevity^26^.

To date, no study has investigated the effect of different species of live yeasts in the juvenile diet of *D. melanogaster* on adult behaviour. To fill this gap, we measured the separate influence of three live yeast species and one inactivated yeast (all added in the juvenile diet) on preimaginal development and on several other fitness-related adult traits. We chose two live yeast species (*Hanseniaspora uvarum, “*HU”; *Metschnikowia pulcherrima* “MP”) based on the fact that they were often detected in the gut of natural *D. melanogaster* populations^20,39^. We also tested live *S. cerevisiae* (“SC”), a yeast species detected at low level in natural *Drosophila* populations, but frequently found on natural food sources^20,40^. We also tested inactivated SC (“drySC”) which is used in many *Drosophila* research laboratories^41^.

## Results

### Preimaginal development

**Figure 1:**
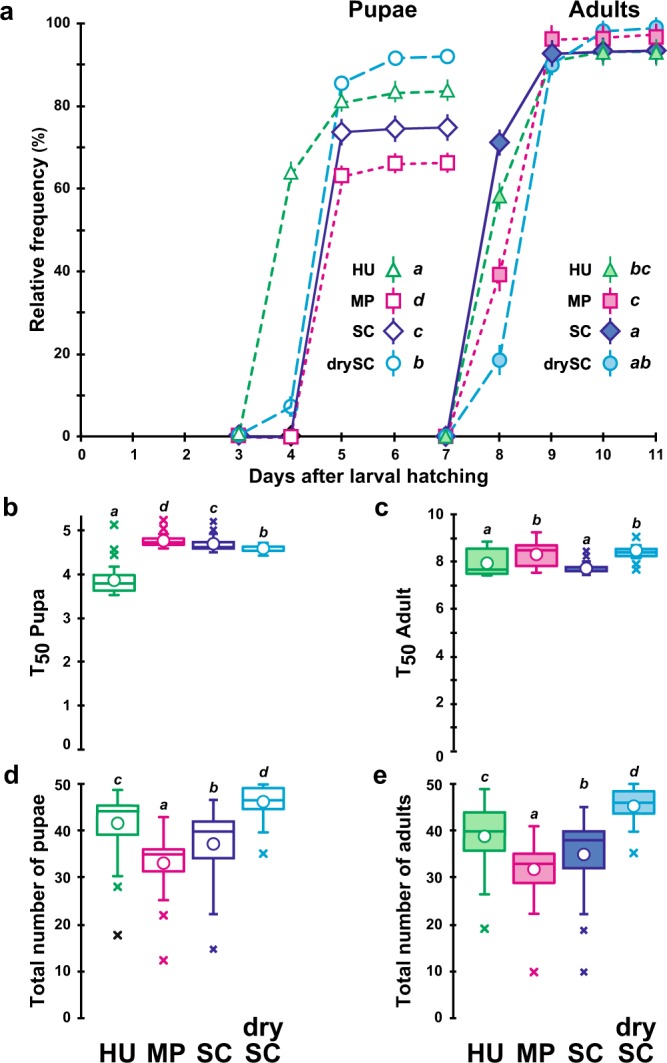
Pre-imaginal development of *Drosophila melanogaster* individuals fed on juvenile diets containing different live yeasts or a dry yeast. (**a**) Relative frequency (in %) of individuals developing into pupa and adult, respectively, as well as respective duration of development (in days) from egg to pupa (empty symbol) or egg to adult (filled symbol) of individuals reared on the four juvenile diets involving one of four alternative yeast treatments, which are represented by different symbols: live *Hanseniaspora uvarum* (HU; green triangle), live *Metschnikowia pulcherrima* (MP; magenta square), live *Saccharomyces cerevisiae* (SC; purple diamond), and dried SC (drySC; cyan circle). Note that adult frequency was calculated based on pupae number. Different italic letters next to the symbol legends indicate significant differences between development curves (Kaplan-Meier procedure with subsequent Log-rank test, and post-hoc multiple comparisons — α = 0.05— with Bonferroni correction). (**b**) Time (in days) at which 50% pupae (T_50_Pupa) (**c**) and 50% adults (T_50_Adult) had developed. (**d**) Total number of pupae (**e**) and adults resulting from 50 eggs raised on each of the four juvenile diets. Box-plots represent the 50% median data (the small horizontal bar indicates the median value, and the white dot represents the mean). The whiskers shown below and above each box represent the first and third quartiles, respectively, and crosses indicate outliers. Different italic letters above whiskers indicate significant differences between means (Kruskal Wallis test with Conover-Iman multiple pairwise comparisons *—*α = 0.05, with Bonferroni correction — (**b**): K_(3df)_ = 89.1, *p* < 10^−4^; (**c**): K_(3df)_ = 50.0, *p* < 10^−4^; (**d**): K_(3df)_ = 70.2, *p* < 10^−4^; (**e**): K_(3df)_ = 69.2, *p* < 10^−4^). *N* = 31–39.

The dynamic of both pupation and adult emergence were affected by juvenile diet (Fig. 1a; Kaplan-Meier procedure with subsequent Log-rank test, Khi^2^_(3df)_ = 441.5, *p* < 10^−4^ and 23.6, *p* < 10^−4^). The transformation of HU-fed larvae into pupae generally occurred one day earlier than in the three other treatments (Fig. 1b; Kruskal Wallis test: K_(3df)_ = 89.1, *p* < 10^−4^). Starting from a constant number of 50 eggs, the number of pupae also strongly varied between diets (46 pupae on drySC diet; 35 pupae on MP diet; Fig. 1d; K_(3df)_ = 70.2, *p* < 10^−4^). The pupa-to-adult development occurred slightly faster in individuals raised on SC and HU diets compared to the two other treatments (Fig. 1c; K_(3df)_ = 50.0, *p* < 10^−4^). The number of emerging flies (Fig. 1e) highly diverged between treatments (46 on drySC; 33 on MP; K_(3df)_ = 69.2, *p* < 10^−4^) but was not different between treatments relatively to the number of pupae (Fig. 1d,e). This indicates that mortality mostly occurred prior to pupation.

### Adult diet preference

**Figure 2:**
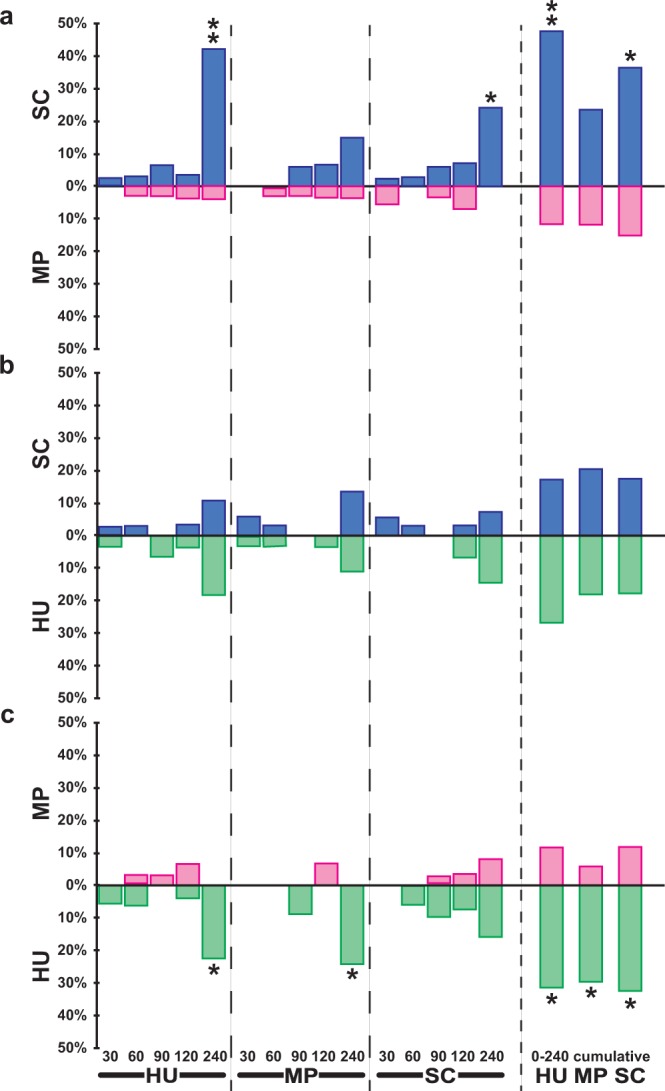
Adult food preference. Single starving male flies were introduced into a Y-maze olfactometer to test their preference to food containing SC yeast (purple bars), MP yeast (magenta bars) or HU yeast (green bars). We tested the three possible dual food choice consisting of (**a**) “SC” *vs*. “MP” food, (**b**) “SC” *vs*. “HU” food, (**c**) “MP” *vs*. “HU” food. The relative frequency of adults choosing either type of diet was noted every 30 min at 30, 60, 90 and 120 min after the test start, and also at 240 min of observation. Therefore, each bar corresponds to a 30-min observation period, except the “240” bar which corresponds to 120 min observation period (between 120 and 240 min). The “0–240 cumulative” bars (shown on the right) present the data pooled between 0 to 240 min. The juvenile diets are indicated below each series of tests. For each choice test, the difference from random was tested with a binomial test (*p < 0.05; **p < 0.01) *N* = 33–34. For other information, please refer to Fig. 1 legend.

In the SC/MP choice test (Fig. 2a), HU- and SC-derived adults showed a clear preference for SC food (47 and 36% after four cumulative hours; binomial test: *p* = 0.005 and 0.047, respectively). Flies subjected to the MP treatment showed no preference. Irrespective of juvenile dietary treatment, adult flies did not show any preference in in the SC/HU choice test (Fig. 2b). In the MP/HU choice test, adults raised on the three juvenile yeast diets containing live yeats (Fig. 2c) showed a constant attraction to HU-rich food which resulted in a strong preference (±30%; binomial test: *p* = 0.042, 0.016 and 0.042, respectively) for this food—or a strong aversion against MP-rich food—after four hours.

### Courtship and copulatory behaviours

All tests involving 5-day old pairs of flies (all pairs are shown as “female × male”) lasted 60 min. All pairs showed a similar courtship intensity (courtship index = CI; Fig. S1a; Kruskal-Wallis test, KW_(15df)_ = 24.05, *p* = 0.064). Their courtship latencies were generally similar (median values: 2–3 min; Fig. S1b; KW_(15df)_ = 29.51, *p* = 0.014), except in MP × HU pairs (MP females × HU males) which showed a delayed latency compared to SC × drySC pairs (SC females X drySC males). The copulation latency (Fig. S1c) was more variable between pairs (KW_(15df)_ = 42.10, *p* = 0.0002). In particular, (*i*) the four pairs involving SC males, (*ii*) drySC males paired with HU or SC females and (*iii*) drySC × MP pairs (drySC females × MP males) copulated faster than drySC × HU pairs (drySC females × HU males).

**Figure 3:**
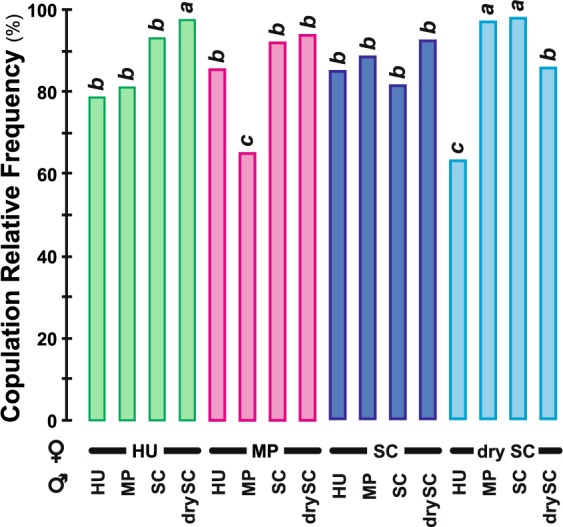
Copulation success rate. The frequency of copulation was measured in female-male pairs during one hour. The juvenile diet treatment for each sex partner (female = upper row; male = lower row) is indicated under corresponding bars. Colors indicate the female juvenile diet (green = HU, magenta = MP, purple = SC, cyan = drySC). Different italic letters above each bar indicate significant differences between copulation frequencies (Wilks *G*^2^ likelihood ratio test completed with a computation of significance by cell—Fisher’s test— G^2^_(15df)_ = 29.8, *p* = 0.013; *N* = 10–29). For other information, please refer to Fig. 1 legend.

The copulation success rate also greatly varied between the 16 pairs according to the diet treatment of both sex partners. The highest copulation frequencies (96.2%) were shown by HU × drySC, drySC × MP and drySC × SC pairs while the lowest ones were observed in MP × MP and drySC × HU pairs (63.2 and 62.1%, respectively; Fig. 3; Wilks *G*^2^ likelihood ratio test, G^2^_(15df)_ = 33.10, *p* = 0.007). No major variation was noted for copulation duration except between HU × MP and MP × MP pairs (18.1 ± 1.0 min *vs*. 14.6 ± 0.8 min, respectively; Fig. S1d; KW_(15df)_ = 26.28, *p* = 0.035).

### Fertility, fecundity and sex ratio

**Figure 4:**
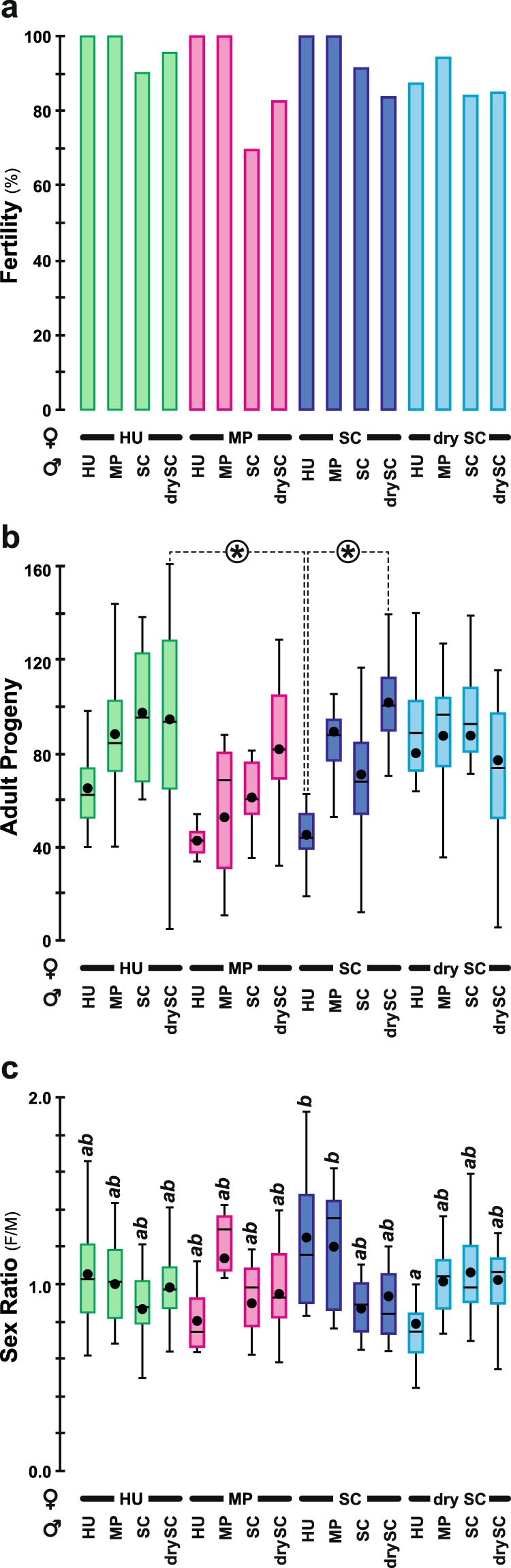
Capacity to leave adult progeny. Using the fly pairs shown in Fig. 3, we measured their (**a**) fertility (ability to leave at least one progeny), (**b**) fecundity (number of adult progenies) and (**c**) sex ratio (daughters/sons) of the progeny. For the three parameters, the juvenile diet of each parent is similar to Fig. 3. *N* = 6–22 (except for MP × HU: *N* = 3). (**a**) Wilks *G*^2^ likelihood ratio test, G^2^_(15df)_ = 17.6, *p* = 0.2863. (**b**) Kruskal Wallis test, K_(15df)_ = 42.3, *p* = 0.0002 — *indicate difference at level α = 0.05. (**c**) Kruskal Wallis test, K_(15df)_ = 27.2, *p* = 0.027. Different italic letters above each bar indicate significant differences between sex ratios. For other information, please refer to Figs 1 and 3 legends.

No fertility difference (ability to leave at least one progeny; Fig. 4a) was noted between pairs (G^2^_(15df)_ = 19.21, *p* = 0.204), but significant differences were found both for fecundity (number of adult progenies; Fig. 4b; KW_(15df)_ = 42.27, *p* = 0.0002) and sex ratio (daughters/sons; Fig. 4c; KW_(15df)_ = 27.23, *p* = 0.027). The pairs either involving HU or SC females with drySC males yielded more adult progenies than SC × HU pairs. Also, the progenies of SC × HU and SC × MP pairs showed a female-biased sex ratio while drySC × HU pairs showed a male-biased sex ratio.

### Longevity

**Figure 5:**
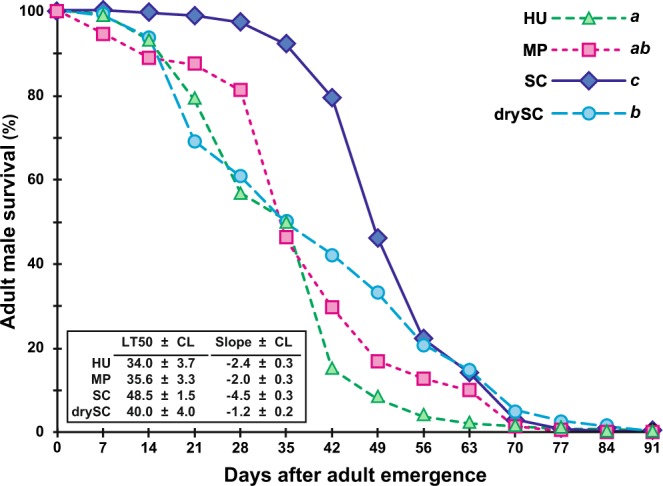
Adult male survival. The survival curves show the frequency of surviving male flies according to time (days) and juvenile diet. Different italic letters next to the symbol legends indicate significant differences between survival curves (Kaplan-Meier procedure with subsequent Log-rank test, and post-hoc multiple comparisons —α = 0.05— with Bonferroni correction). The inset shows the estimated time (in days ± 95% confidence limits) at which 50% flies were dead (lethality time 50% = LT50) and the lethality slope (number of flies dying per day). *N* = 15 groups of flies. For other information, please refer to Fig. 1 legend.

Adult male survival diverged between the four juvenile diets (Fig. 5; Kaplan-Meier procedure, Khi^2^_(3df)_ = 90.8, *p* < 10^−4^). In particular, the death of SC flies occurred later (LT50 = 48.5 ± 1.5 days) compared to other flies (LT50 = 34.0 ± 3.7 – 40.0 ± 4.0 days) but their death rate (slope) was steeper (4.5% *vs*. 1.2–2.5% per day, respectively). The last surviving flies reached a similar age (10–12 weeks) in all treatments.

### Cuticular hydrocarbons

**Figure 6:**
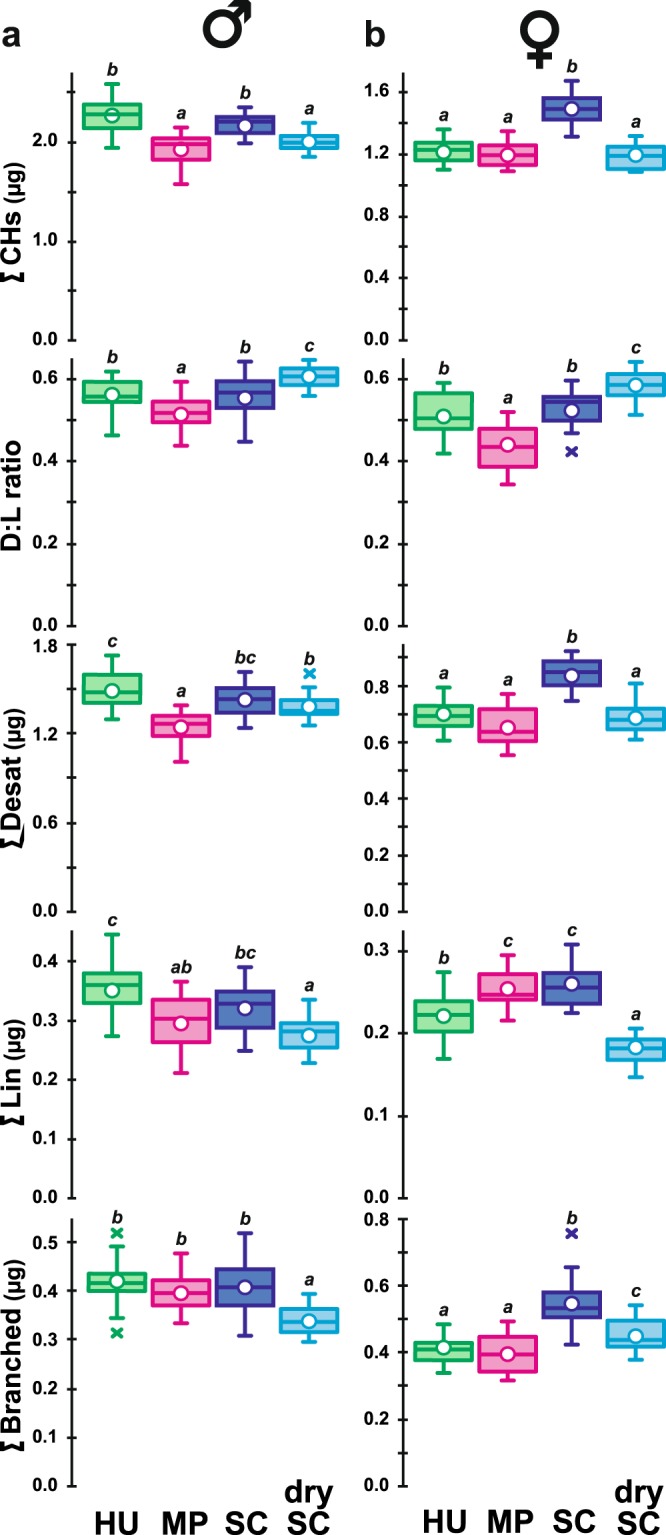
Cuticular hydrocarbons in mature adults. The principal cuticular hydrocarbons (CHs) measured in µg per fly are shown in 5-day old male (**a**) and female flies (**b**). From top to bottom, data shown correspond to the total absolute amount of CHs (∑CHs), the ratio between Desaturated and Linear saturated CHs (D:L ratio). Below we show the sums of desaturated CHs (∑Desat), of linear saturated CHs (∑Lin) and of branched CHs (∑Branched). Different italic letters above whiskers indicate significant differences between means (Kruskal Wallis test with Conover-Iman multiple pairwise comparisons *—*α = 0.05, with Bonferroni correction; all tests are significant at level *p* = 10^−4^: K_(3df)_ = 36.7 & 44.7 for ∑CHs; K_(3df)_ = 32.6 & 42.2 for D:L ratio, K_(3df)_ = 38.0 & 42.9 for ∑Desat; K_(3df)_ = 26.3 & 49.2 for ∑Lin; K_(3df)_ = 28.0 & 38.9 for ∑Branched, for male and female flies respectively). *N* = 20 for each diet and sex. For other information, please refer to Fig. 1 legend.

Juvenile diet influenced the levels of the three principal CH groups in both sexes: desaturated CHs (∑Desat; KW_(3df)_ = 38.01 and 42.85—both *p* < 10^−4^—in males and females, respectively), linear saturated CHs (∑Lin; KW_(3df)_ = 26.30 and 49.18— both *p* < 10^−4^—in males and females, respectively) and branched CHs (∑Branched; KW_(3df)_ = 28.01 and 38.92— both *p* < 10^−4^; Fig. 6). These variations affected the overall CHs production (∑CHs; KW_(3df)_ = 36.70 and 44.71—both *p* < 10^−4^) and the ratio between ∑Desat and ∑Lin (D:L ratio; KW_(3df)_ = 32.56 and 42.23—both *p* < 10^−4^). In particular, HU males and SC male and female flies showed higher ∑CHs (2265 ± 40, 2169 ± 35 and 1381 ± 20 ng, respectively) than same-sex flies raised on the two other diets (1937 ± 35 and 1998 ± 25 ng in males and 1115 ± 17 and 1098 ± 125 ng in females). Also, MP flies showed the lowest D:L ratio (0.51 ± 0.01 in males and 0.44 ± 0.01 in females) while drySC flies showed the highest ratio (0.61 ± 0.01 and 0.58 ± 0.01 in males and females respectively).

## Discussion

While several studies reported the possible influence of microorganisms on various *Drosophila* mating and reproductive aspects, very few investigated life-long effects of live yeasts only provided in the juvenile diet. We found that each of the three live yeasts (*Hanseniaspora uvarum, “*HU”; *Metschnikowia pulcherrima* “MP”; *S. cerevisiae* yeast, “SC” (and the inactivated SC = “drySC”) induced distinct effects on pre-imaginal development and on several adult life traits: courtship, copulation, fertility, fecundity and cuticular hydrocarbons.

### Preimaginal development

First, preimaginal development was affected. In particular, the HU juvenile diet induced a faster egg-to-pupa—but not egg-to-adult—development, while the SC diet induced a slightly faster pupa-to-adult development, compared to the other diet treatments. The similar proportion of adults developing from pupae in each treatment indicates a very reduced effect of the yeast diet on pupal survival. Differently, egg-to-larva survival (assessed on the ability of fertilized eggs to transform into pupae) was strongly affected by the juvenile diet. The much higher proportion of pupae and adults developing on drySC diet, compared to MP diet indicates that the wild-type strain used in this study is adapted to drySC yeast with regard to larval development. The decreased proportion of MP-fed pupae indicates that MP-rich diet induced higher larval lethality compared to SC- and HU-rich diet. The deleterious effect of MP on larval development was already described^37,42^. It may be caused by pulcherrimic acid depleting the amount of iron available in the food, this affecting the growth of bacteria and fungi composing the fly microbiome^43,44^. Conversely, some yeasts can induce developmental advantage such as *Pichia kluyveri* in *D. melanogaster*^25^, or *Candida* sp. and *M. pulcherrima* in *D. suzukii*^42^. Therefore, if the mechanisms underlying developmental success vary between *Drosophil*a species, they could partly depend on the quality of nutrients—such as essential amino acids—provided by each type of yeast, and/or on the yeast-bacteria interaction^12,45–47^. We cannot exclude the alternative possibility that the three live yeasts grew at different rates thus implying that they were more abundant in some diet treatments than in others. However, we were unable to measure and compare the growth of yeast in the media as well as the amount of yeast ingested by larvae during their development.

### Food preference

Adult male food preference was clearly altered by the live yeast species added in their juvenile diet. HU- or SC-rich diets were generally preferred over MP-rich diet while the HU/SC choice induced no preference. Adult food preference may result from early developmental depletion in essential amino acids together with the activation of chemosensory neurons responding to yeast volatile molecules^47–49^. If HU-rich food attracted *D. melanogaster* flies, it repulsed tephritid fruit flies^50^, thus indicating that the same yeast can induce species-specific response. Varying quality of the same yeast species, e.g. SC, may also affect adult preference: some of us previously found that adults raised on food containing live SC preferred live SC over drySC, whereas adults raised on food containing drySC, heatedSC or no SC at all, did not show any preference^26^. Our present data suggest that food preference does not result from a learning effect since flies exposed to a given diet did not show preference for similar food. We rather believe that early developmental ingestion of certain yeast species (HU and, to a lesser extent, SC) promoted a wild-type like growth, guidance and arborization of the neurons involved in chemosensory perception^5,12,51^. However, food preference could also result from early larval exposure to species-specific metabolites (by-products of the transformation of food molecules by gut-associated yeasts) such as acetic acid, 3-methyl-1-butanol or 2-phenyl-ethanol which can change larval and adult responses to similar metabolites^24,29,30^. The variation of adult behaviour may also be partly due to the persistence of live yeasts remaining in the gastrointestinal tract through the entire metamorphosis, as previously found, using live GFP-labelled SC strains^26^. However, the unavailability of fluorescent marker strains of HU and MP prevented us to further test this hypothesis. Moreover, both the temperature and the quality of food can change *Drosophila* appetence for yeast^52,53^. While the quantitative increase of dietary yeast led to a huge increase in the total abundance of gut microbes, it also caused a major depletion of their diversity in the *Drosophila* gut ecosystem^21^.

### Mating behaviours

The courtship and copulation experiments did not reveal consistent responses given that none of the juvenile dietary treatments induced a uniform effect on either sex independently of the dietary treatment of the mating partner. This suggests that the behavioural responses shown by each pair resulted from a complex interaction between the two mating partners. Courtship parameters were very similar between pairs while copulation parameters frequently varied. Our data indicate that SC—and to a lesser extent drySC—juvenile diet tended to promote faster copulation. The significantly different copulation duration between MP × MP (MP females × MP males) and HU × MP (HU females × MP males) pairs indicates that juvenile diet influences female copulation duration, at least with MP males. Moreover, the pairs either showing delayed copulation latency (drySC × HU) or short copulation duration (MP × MP) also had the lower copulation success rate. Differently, three other pairs (drySC × SC, drySC × MP and HU × drySC) showed an increased copulation success rate. Therefore, the presence of SC (live or dry) in the juvenile diet tended to enhance copulation ability in either sex, depending on the diet treatment of the sex partner. These data again suggest that increased copulatory performances result from an adaptative process after many generations of adaptation on drySC-rich food in the laboratory, as reported for other diets^54,55^. The fact that SC-rich diet also induced enhanced reproductive behaviour suggests that the nutriments contained in both SC and dry SC yeasts participate in this adaptative process. Indeed, several studies showed that different amino acids (produced in different combinations by different yeast species) could affect fly chemosensory ability (similarly to the effect found on food preference)^12,47,51^.

### Fertility, fecundity and sex ratio

The comparison between SC × HU (SC females × HU males) *vs*. SC × drySC (SC females × drySC males) pairs suggests that the juvenile diet also affects male fecundity. This result could also reflect a long-term adaptation of the wild type strain to laboratory drySC diet. However, we do not know whether the variation in fecundity was caused by an increased number of progeny (in SC × HU pairs), or an increased of preimaginal mortality (in SC × drySC pairs). On the one hand, an increased progeny could be a direct consequence of the amino acid content (due to the yeast present in the juvenile diet) influencing the quality and quantity of gametes produced and/or the number of eggs laid^45,56^. On the other hand, an increased lethality could be caused by a lower level of gametic compatibility between the two sex partners according to their respective juvenile diet as described in *Callosobruchus maculatus*^57^.

The sex ratio examination provides a complementary angle to answer this question: SC × HU and SC × MP pairs yielded an increased proportion of daughters while drySC × HU pairs had more sons. Following the dual hypothesis proposed above, the sex ratio variation would either result from (*i*) the differential ability of x- and y-bearing sperm cells to fertilize oocytes, or (*ii*) a sex-biased mortality. The second hypothesis may be valid for the SC × HU pairs which produced fewer adult progeny, which might have been caused by an excessive death of sons given the female-biased sex ratio. The comparison between SC × HU *vs*. drySC × HU pairs also indicates that the quality of SC yeast in the juvenile diet of females can influence the sex ratio of their progeny.

The simultaneous examination of pre- and postzygotic reproduction-related characters highlights yeast species-specific effects in the juvenile diet. First, a relationship was detected between copulation latency and fecundity: drySC males paired with HU or SC females copulated faster and produced more abundant progeny than all other pairs. Differently, HU- and drySC-rich diet induced reciprocal effects in the two sexes: HU × drySC pairs showed both the highest copulation success rate and adult progeny, while drySC × HU pairs showed both the lowest copulation success rate and proportion of daughters. Also, HU-rich diet generally promoted high fertility in both sexes and high female—but not male—fecundity (see Fig. 4a,b) while drySC diet promoted high male fecundity. The low fecundity shown by SC × HU pairs may be explained by the low fecundity of HU-fed males.

### Cuticular pheromones and longevity

Some cuticular hydrocarbons (CHs) acting as mating stimuli could be involved in the variation of the copulatory performance. While the overall level of CHs was shown to depend on the presence of specific bacteria of the microbiome^15^, our data do not fully support this hypothesis. The discrepancy between the two data sets may be explained by several experimental differences: we tested single pairs of flies raised only during preimaginal development on food enriched with live yeast while the previous study assessed multiple-choice mating in flies raised on bacteria-free food (after antibiotic treatment) during their entire life. This may explain why the CH variations resulting from yeast variation in the juvenile diet were less dramatic than those induced by the antibiotic treatment^15^. It is worth to note that while the juvenile yeast diet treatment changed the two main CH parameters (∑CHs; D:L ratio) in both sexes in a similar manner, CHs groups differently varied between the sexes.

Two previous studies revealed a negative relationship between the level of desaturated CHs (∑Desat) and longevity^9,26^. Our results do not show this negative relationship. Since SC-fed male flies tested here did not show an overall increased longevity as the flies tested in the two previous studies, the negative relationship between the level of desaturated CHs and longevity may not have been detected. In fact, while SC-rich diet allowed about 50% male flies to live two weeks longer than in the other treatments, once these flies started to die, their lethality rate (4.5% per day) was faster than in other treatments (1.2–2.5% per day). This indicates that SC-fed flies showed a more homogeneous longevity compared to other flies.

In summary, feeding larvae with a diet containing different live yeasts as well as dry SC yeast, strongly affected several adult characters related to fitness. Although the biological mechanisms underlying and linking these effects remain unknown, our study suggests that the species of live yeast(s) found and ingested by *Drosophila* on natural food sources induces dramatic consequences on their development, food search, reproduction and survival. Given that natural food sources generally host multiple yeast species, this implies that their ingestion could induce much more complex effects with regard to the development and fitness of saprophagous and microbivorous insects.

## Material and Methods

### Yeast strains and food preparation

We used three live yeast species and one inactivated yeast species. The live yeast species were the *Saccharomyces cerevisiae* (SC) wild type strain BY4742 (MATα his3Δl leu2Δ0 lys2Δ0 ura3Δ0) (EUROSCARF, Frankfurt, Germany), the *Hanseniaspora uvarum* DCB T6 211 strain (HU) isolated from grape must^58^, and the *Metschnikowia pulcherrima* DS T0 V15 strain (MP). These live yeast species were chosen based on their natural abundance either in the digestive tract (HU and MP) or on many food sources of *D. melanogaster*. The inactivated yeast species was a wild type SC strain produced by vacuum concentration and inactivated by spray drying (drySC; E50, Lesaffre Culinary Strasbourg, France). We chose to also test drySC, since it is widely used as food medium in Drosophila research laboratories. Thus, all in all, four different juvenile diets were tested, three containing the live yeast species SC, HU and MP, and one containing dry SC.

Pre-cultures for the juvenile diet containing live yeast species were set up over 48 h on Yeast extract (10 g/l)-Peptone (20 g/l)-Dextrose (20 g/l) medium (YPD) at 25 °C with rotation at 250 rpm with each yeast colony in an Erlenmeyer flask containing 100 ml of sterile YPD. An adequate volume of the pre-culture was then transferred to an Erlenmeyer flask containing 100 ml of fresh YPD medium to reach 0.05 OD_600_. The culture was incubated at 25 °C with 250 rpm rotation to allow growth to the early stationary phase. All juvenile food media contained corn flour (65.4 g/l) and agar (9.2 g/l) to be mixed with live yeasts (adjusted to 5 × 10^8^ yeast cells/ml of food corresponding to 65 g/l yeast dry weight; this estimation is based on the theoretical weight of individual yeast cells). Prior to adding the live yeast species to the corn flour-agar-medium, the medium was allowed to cool down to a temperature of around 30 °C. All food types were used within 48 h of production. We did not quantify the growth of the different live yeast species on the corn flour-agar-medium. Due to its lower amount of moisture, the juvenile diet with drySC may have contained a higher percentage of protein at least at the beginning of the experiment (larval development), even though not at the end of the experiment, given that drySC did not grow.

### *Drosophila* culture and egg collection

*D. melanogaster* strains were raised in 150 ml glass vials containing 50 ml of drySC/cornmeal/agar medium and maintained in a breeding room at 24.5 ± 0.5 °C with 65 ± 5% humidity on a 12:12 h light/dark cycle (subjective day from 8:00 am to 8:00 pm). Flies were transferred every two days to avoid larval competition and to regularly provide abundant progeny for testing. We used Dijon2000 (Di2), a wild-type strain maintained in our lab for almost two decades. Young emerging flies were housed in groups of 50–100 individuals to allow mating in a 150 ml vial containing 50 ml of plain food (regular laboratory food with drySC). After 2–3 days, adults were transferred to a fresh food vial for 3–4 h to allow egg laying. For each collection, we used 8–12 vials from which eggs were collected.

### Pre-imaginal development

Eggs were washed three to four times in distilled water and transferred with a fine sterilized brush (washed in 99% ethanol prior to each series of egg transfer) in groups of 50 to a 150 ml vial containing 50 ml of each type of juvenile diet (HU, *N* = 39; MP, *N* = 37; SC, *N* = 37; drySC, *N* = 32). In each vial, we measured the duration of development by scoring twice a day the number of newly formed (white) pupae and of newly emerged male and female adults. All emerging adults were transferred into 150 ml vials with the regular lab medium (“drySC” food) for further testing. Adults were maintained in same-sex groups of 8–12 flies except in copulation and food preference tests where males were kept isolated.

### Food preference

5-day old males were individually maintained during the 24 h preceding the test in an empty vial containing only a humid filter paper (diameter = 2 cm). Tests were always performed between 8 am and noon. After a brief anaesthesia on ice, each male was introduced into a Y-shape olfactometer consisting of a straight arm (where the fly was introduced) divided in two arms. Each of these two arms contained a filter paper (diameter = 2 cm) previously incubated at 25 °C for 30 min with one of two types of food medium (SC *vs*. MP; SC *vs*. HU; MP *vs*. HU). Excess food was removed with a spatula cleaned with 90% ethanol. The test was performed, at 24.5 ± 0.5 °C, during a 240-min period under far-red light (LED bulbs). After the test start, the position of the fly in the olfactometer was noted every 30 min until 120 min and finally at 240 min. Flies were rarely seen retreating into the straight arm of the device. Flies from different diet treatments were simultaneously tested. Tests involving different diet-fed individuals and dual food choices were performed over several days to randomize uncontrolled experimental variation. *N* = 34, except HU-derived males for SC *vs*. MP test (*N* = 33).

### Reproduction

We measured the copulatory ability in pairs of 5-day old male and female flies. Tests were performed between 9 am and noon, for 60 min under white light, at 24.5 ± 0.5 °C. One male was aspirated (without anaesthesia) and released into a small plexiglas observation chamber (1.6 cm^3^). After 10 min, a virgin female was introduced. During the first 10 min (or until copulation occurred), we observed the male activity and noted both the courtship latency (time from introduction to time of behavioural onset) and the courtship index (CI = percentage of time spent by the male courting the female during 10 min or until copulation occurred). Pairs were kept together for 60 min to estimate overall copulation frequencies. According to the pairs, *N* = 10 to 29. Beside the copulation latency (time from introduction to time of copulation onset), we calculated the cumulative proportion of flies mating during the observation period (copulation success rate) and the duration of copulation events (time between copulation onset and separation). Females copulating within the 60 min observation period were transferred alone to a fresh food vial (the male was immediately discarded), while non-mating pairs were individually kept in a food vial (and the male discarded 24 h later). In all cases, the presence (fertility), number (fecundity) and sex ratio (female: male) of adult progeny were noted between 12 and 16 days after the mating test. Tests involving males and females subjected to different diet treatments were mixed each day and performed over several days to randomize uncontrolled experimental variation.

### Longevity

Adult longevity was only determined in males since (*i*) we did not control for the mating status of females and (*ii*) the presence of progeny affects female survival. Males held in small groups of 8–12 flies were transferred every week to fresh food vials. The number of surviving males was scored at each transfer. To maintain the group size as constant as possible, we pooled flies from vials with a high mortality. Two measures of lethality were recorded: LT50 (time at which 50% of the flies had died) and lethality slope (the steepness of this curve indicating the proportion of flies dying per day). Initial sample size were about equal: N = 182 (HU), 178 (MP), 173 (SC) and 174 (drySC).

### Cuticular hydrocarbons

5-day old flies were frozen for 5 min at −20 °C and individually extracted for 5 min at room temperature using 30 µl of a mixture of hexane and methylene chloride (50/50; vol/vol) to extract a large variety of compounds. The solution also contained 3.33 ng/µl of C26 (*n*-hexacosane) and 3.33 ng/µl of C30 (*n*-triacontane) as internal standards. Cuticular hydrocarbons (CHs) were quantified by gas chromatography using a Varian CP3380 gas chromatograph fitted with a flame ionization detector, a CP Sil 5CB column (25 m by 0.25-mm (internal diameter); 0.1 µm film thickness; Agilent) and a split–splitless injector (60 ml/min split-flow; valve opening 30 sec after injection) with helium as the carrier gas (50 cm/sec at 120 °C). The temperature program began at 120 °C, ramping at 10 °C/min to 140 °C, then ramping at 2 °C/min to 290 °C and holding for 10 min. Individual CH profiles were determined by integration of 46 peak areas in males and females, representing all of the peaks that could consistently be identified in all individuals^59^. The chemical identity of the peaks was determined using gas chromatography–mass spectrometry system equipped with a CP Sil 5CB column. The amount (µg/insect) of each component was calculated based on the readings obtained from the internal standards. For the sake of clarity we only show the principal CH groups: desaturated CHs (∑Desat), linear saturated CHs (∑Lin) and branched CHs (∑Branched). We also show the overall CHs sum (∑CHs) and the ratio between ∑Desat and ∑Lin (D:L ratio). This ratio was calculated using the formula ([D − L]/[D + L]). Twenty flies were tested per sex and treatment.

### Statistics

All statistical analyses were performed using XLSTAT 2019^60^.

Developmental and male survival curves were compared using the Kaplan-Meier procedure with a subsequent Log-rank test and a post-hoc multiple pairs comparison (α = 0.05 with Bonferroni correction). In addition, for each diet, logistic regression was used to characterize the relationship between development and time by estimating the time duration (in days) at which 50% pupae (T_50_Pupa) (**c**) and 50% adults (T_50_Adult) had developed together with their corresponding regression slope^61^. Thereafter, we carried out an inter-diet comparison for these two parameters using a Kruskal-Wallis test with Conover-Iman multiple pairwise comparisons (α = 0.05, with Bonferroni correction) after excluding extreme outliers using Tukey’s method^62^.

The number of pupae and adults, the D:L ratio and ∑CHs, the total number of adult progeny and the sex ratio were also compared using the Kruskal-Wallis test. Frequencies were compared using either a binomial test (pair comparisons, food preference) or a Wilks *G*^2^ likelihood ratio test completed with a computation of significance by cell (Fisher’s exact test).

## Supplementary information

Supplemental Figure 1.

Murgier raw dataset

## Data Availability

A xlsx File containing all raw data is available (MURGIER_data.xlsx).
