# Supplementary material for: Live yeast in juvenile diet induces species-specific effects on Drosophila adult behaviour and fitness
Source: Sci Rep. 2019 Jun 20;9:8873. doi: 10.1038/s41598-019-45140-z (PMC6586853; doi:10.1038/s41598-019-45140-z)
Supplement: Supplementary file 1 — Supplemental Figure 1. [file 41598_2019_45140_MOESM1_ESM.pdf]

Live yeast in juvenile diet induces species-specific effects on Drosophila adult behavior and fitness

Juliette Murgier, Claude Everaerts, Jean-Pierre Farine, Jean-François Ferveur

SUPPLEMENTARY INFORMATION

Supplemental Figure 1. Courtship and copulation parameters. In the pairs tested for copulation frequency (see Figure 3), we also measured during the first 10 min (or until copulation occurred), the male activity and noted both (**a**) the courtship latency (time in min from introduction to time of behavioral onset) and (**b**) the courtship index (percentage of time spent by the male courting the female during 10 minutes or until copulation occurred). Pairs were kept together for 60 minutes to estimate their overall copulation frequencies (Figure 3). (**c**) Beside their copulation latency (time in min from introduction to time of copulation onset), we determined the duration of copulation (**d**; time in min between copulation onset and separation). The pre-adult diet treatment for each sex partner (female=upper row; male=lower row) is indicated under corresponding bars and box-plots. Different letters indicate significant differences. No difference was found for "courtship index" and only two pairs significantly varied for "courtship latency" and "copulation duration". More differences were found for "copulation latency". Data were compared using the Kruskal Wallis test (**a**: $K_{(15df)}=29.5$, $p=0.014$; **b**: $K_{(15df)}=24.1$, $p=0.064$; **c**: $K_{(15df)}=42.1$, $p=0.0002$; **d**: $K_{(15df)}=26.3$, $p=0.035$). $N=10\text{-}24$. For other informations, please refer to Figures 1 and 3 legends.

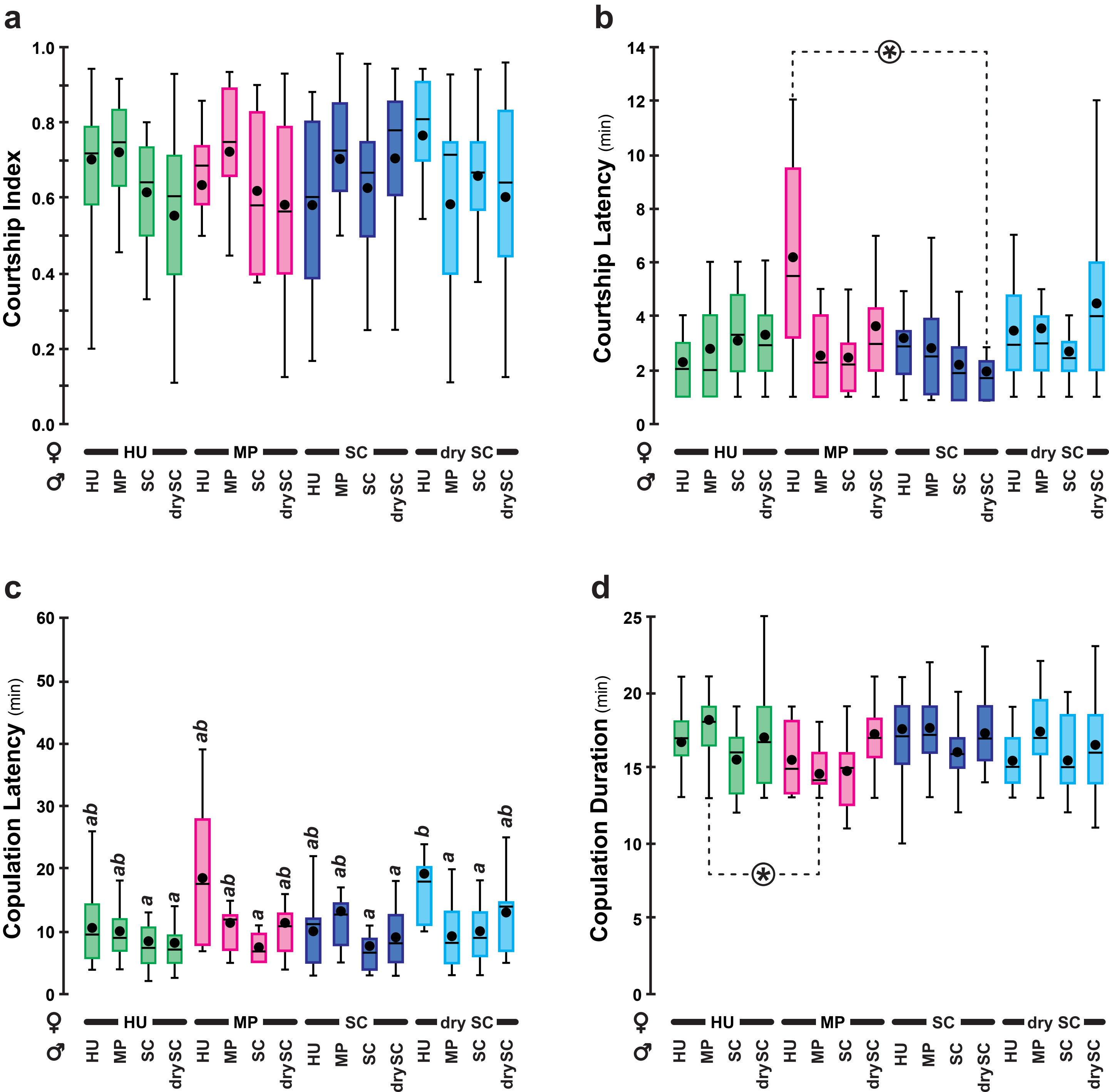
a
Courtship Index
1.0
0.8
0.6
0.4
0.2
0.0
b
Courtship Latency (min)
14
12
10
8
6
4
2
0
c
Copulation Latency (min)
60
50
40
30
20
10
0
ab ab a a ab ab a ab ab ab a a b a a ab
d
Copulation Duration (min)
25
20
15
10
5
0
♀ HU MP SC dry SC
♂ HU MP SC drySC HU MP SC drySC HU MP SC drySC HU MP SC drySC
